# Spatially multicellular variability of intervertebral disc degeneration by comparative single‐cell analysis

**DOI:** 10.1111/cpr.13464

**Published:** 2023-04-06

**Authors:** Peng Lin, Pulin Yan, Jun Zhu, Sha Huang, Zhong Wang, Ou Hu, Huaijian Jin, Yangyang Li, Liang Zhang, Jianhua Zhao, Lin Chen, Bing Liu, Jian He, Yibo Gan, Peng Liu

**Affiliations:** ^1^ Department of Spine Surgery, Center of Orthopedics, State Key Laboratory of Trauma, Burns and Combined Injury, Daping Hospital Army Medical University (Third Military Medical University) Chongqing 400042 China; ^2^ Center of Bone Metabolism and Repair, State Key Laboratory of Trauma, Burns and Combined Injury, Trauma Center, Research Institute of Surgery, Laboratory for the Prevention and Rehabilitation of Military Training Related Injuries, Daping Hospital Army Medical University (Third Military Medical University) Chongqing 400042 China; ^3^ State Key Laboratory of Proteomics, Academy of Military Medical Sciences Academy of Military Sciences Beijing 100071 China; ^4^ State Key Laboratory of Experimental Hematology, Institute of Hematology Fifth Medical Center of Chinese PLA General Hospital Beijing 100071 China; ^5^ Key Laboratory for Regenerative Medicine of Ministry of Education Institute of Hematology, School of Medicine, Jinan University Guangzhou 510632 China; ^6^ State Key Laboratory of Experimental Hematology Institute of Hematology and Blood Diseases Hospital, Chinese Academy of Medical Sciences Tianjin 300020 China; ^7^ Laboratory of Basic Medicine The General Hospital of Western Theater Command Chengdu 610031 China

## Abstract

Previous studies have revealed cellular heterogeneity in intervertebral discs (IVDs). However, the cellular and molecular alteration patterns of cell populations during degenerative progression remain to be fully elucidated. To illustrate the cellular and molecular alteration of cell populations in intervertebral disc degeneration (IDD), we perform single cell RNA sequencing on cells from four anatomic sites of healthy and degenerative goat IVDs. *EGLN3* ^+^ StressCs, *TGFBR3* ^+^ HomCs and *GPRC5A* ^+^ RegCs exhibit the characteristics associated with resistance to stress, maintaining homeostasis and repairing, respectively. The frequencies and signatures of these cell clusters fluctuate with IDD. Notably, the chondrogenic differentiation programme of *PROCR* ^*+*^ progenitor cells is altered by IDD, while notochord cells turn to stemness exhaustion. In addition, we characterise *CAV1* ^+^ endothelial cells that communicate with chondrocytes through multiple signalling pathways in degenerative IVDs. Our comprehensive analysis identifies the variability of key cell clusters and critical regulatory networks responding to IDD, which will facilitate in‐depth investigation of therapeutic strategies for IDD.

## Graphical abstract

Overview of cell alteration in IDD. The key biological events of IDD were revealed by scRNA‐seq analysis, including variability of chondrocytes, switch of PC fates and vessel penetration.

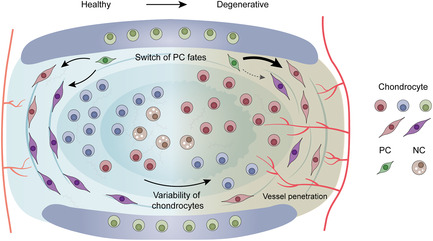

## 1 INTRODUCTION

Degenerative disc disease (DDD) is one of the main causes of lower back pain. Over 90% of the elderly suffer from DDD, which brings a huge burden on the global health system.^1^ At present, the main treatment of DDD is palliative care to relieve the symptoms, which leads to relapse and various complications.^2^ However, the insufficient analysis of the pathological mechanism of DDD is an obstacle to find effective therapeutic approaches. Therefore, an in‐depth understanding of the mechanisms driving disc degeneration is of critical importance.

Intervertebral disc (IVD) is composed of four compartments, nucleus pulposus (NP), inner annulus fibrosus (IAF), outer annulus fibrosus (OAF), and cartilage endplate (CEP).^3^, ^4^ The pathology of intervertebral disc degeneration (IDD) is multifactorial and driven by progressive dysfunction. The initial proteoglycan loss results in NP dehydration, accompanied by AF fissures and CEP ossification.^5^, ^6^, ^7^, ^8^, ^9^ The damages to structure contribute to the disorder of IVD mechanics and lead to severe symptoms in the end, motivating the search for underlying cellular and molecular mechanism. In recent years, single cell RNA sequencing (scRNA‐seq) showed advantages in dissecting IVD physiology and pathology.^10^, ^11^, ^12^, ^13^, ^14^, ^15^, ^16^, ^17^ However, due to the inevasible individual difference and the limitation of specimen accessibility in human studies, a comprehensive interpretation of the hierarchical mechanisms underlying the cellular and molecular alteration during disc degeneration still needs to be claimed. Thus, spatially resolving cell heterogeneity and its variation using an appropriate animal model of IDD would be a critical enabler for further dissecting the intricacies of IDD mechanism.

Goat is an ideal model animal to study degenerative progression and establish parallels with humans. Compared with human specimens, goat IVDs are accessible with distinguishable anatomic structures. Compared with mice and rats, goat IVD shows its advantage of similar anatomy and biomechanics to human IVD and sufficiency in cell abundance for scRNA‐seq.^18^ By setting self‐controlled experiments, individual differences and batch effects can be mostly eliminated. Thus, we established goat IDD model by needle puncture and performed unbiased scRNA‐seq on NP, IAF, OAF, and CEP, respectively, to analyse the variation in cell composition and molecular characteristics between healthy and degenerative IVDs. Our study reveals the spatial cellular and molecular variability associated with degeneration and provides important clues for the pathological mechanism of DDD.

## 2 MATERIALS AND METHODS

### 2.1 Construction of IDD models in goat

Five skeletally mature male Chinese mountain goats, approximately 3‐year‐old and weighting between 35 and 50 kg, were used for this study. Goat skeletal maturity was confirmed by radiographs showing closure of the distal femoral and proximal tibial growth plates.^19^ The goats were housed in the air‐conditioned and dark–light cycle‐controlled pens and were cared for by a qualified veterinarian during the whole study.

To establish a degeneration model of IVD, goats were anaesthetised via inhalation of isoflurane, and then intubated and maintained on an isoflurane‐oxygen mixture throughout the surgical procedure. Using standard aseptic technique, the lumbar IVDs were exposed via an open, left lateral retroperitoneal, and translocation approach. The disc spaces were identified and counted using lateral fluoroscopy and a titanium Kirschner wire was placed in the L1 or L2 vertebral body as a fiducial marker to enable the identification of vertebral levels on radiographs. Three randomly selected samples among L1‐L2, L2‐L3, L3‐L4, L4‐L5 and L5‐L6 discs were injured to generate degenerative IVDs using a drill bit with 4.5 mm in diameter to insert 15 mm and rotated 360°.^18^ The two other uninjured discs of the same goats were used as the blank control. The surgical incision was then closed in layers, and goats were hand‐recovered by veterinary staff until ambulatory, upon which they were returned to standard housing. Perioperatively, animals were administered with transdermal fentanyl (2.5 mcg/kg/h) and intravenous flunixin meglumine (Banamine, 1.1 mg/kg) for analgesia. Florfenicol (40 mg/kg) was administered for antimicrobial prophylaxis. Two months after injury, the spines were harvested *en bloc* from the goats until euthanasia via an overdose of isoflurane for further analysis.

### 2.2 7.0T Micro‐magnetic resonance imaging (MRI) of IVD sample

The dissected goats' functional spinal units were stored in the MACS tissue storage solution (130–100‐008, Miltenyi Biotec) at 4°C for 1 h before scanning. Then, T2‐weighted and T1‐weighted sagittal and axial scanning were performed using a 7.0T micro‐MRI machine (BioSpec 70/20 USR, BRUKER) according to the instructions from a previous study.^20^ A blinded observer evaluated the grade of degeneration on T2‐weighted scans based on the Pfirrmann grading system.^21^

### 2.3 Preparation of goat IVD single‐cell suspension

To avoid the obstructed dissociation caused by dense ECM and capture rare cell populations, we used a cocktail of ECM‐specific enzymes within a shorter time to improve the isolation efficiency and minimise the damage to the embedded cells, then applied magnetic bead separation to remove the dead cells and tissue debris.^22^ In detail, the disc sample was stored in the MACS Tissue Storage Solution at 4°C within 4 h before digestion. Segregated NP, IAF, OAF, and CEP samples were chopped as finely as possible and washed repeatedly with phosphate‐buffered saline (PBS) until there was no visible blood contamination. We prepared single‐cell suspensions according to the previous study.^23^ The samples were transferred to pre‐warmed TrypLE Express (SH30042.01, Hyclone) at 37°C for 30 min, and digested with 0.2% pronase (10165921001, Roche) for 60 min. After that, depending on the residue, the tissues were incubated with 0.2% collagenase II (C6885,Sigma‐Aldrich) with gentle rotation for 2–4 h. The enzymatic digestion is terminated when the tissue pieces have entirely decomposed. The single cells were filtered through a 40‐mm cell strainer (JETBIOFIL) to remove tissue debris. The single cell suspension was then treated with the 1× red blood cell lysis solution (130–094‐183, Miltenyi Biotec) to remove the residual blood cells. We eliminated dead cells by labelling cells with Dead Cell Removal Kit (Miltenyi Biotec) and removing them over an LS column in the magnetic field of a MidiMACS™ Separator (Miltenyi Biotec). Cell viability was measured by AO/PI staining using the Rigel S2 Cell Counter (Countstar).

### 2.4 Library construction for scRNA‐seq

The cells were washed with PBS three times and concentrated to 700–1200 cells/μL. The suspension was then loaded on a Chromium Controller (10× Genomics) for scRNA‐seq library construction The libraries were sequenced on an Illumina X‐Ten sequencing platform to generate 150‐bp paired‐end reads, according to the manufacturer's instructions (Berry Genomics).

### 2.5 scRNA‐seq data pre‐processing

Raw reads were aligned to the goat genome (*Capra hircus*, version 100), and gene expression matrices were generated for each sample by the Cellranger (version 4.0.1, 10x Genomics). Python package Scanpy (v1.9.1) workflow was used for data integration and dimensionality reduction and clustering.^24^ In short, the BBKNN function was used to remove batch effects among the datasets. UMAP (uniform manifold approximation and projection) was used for dimensionality reduction and cell clusters were defined using leiden algorithm.

### 2.6 Differentially expressed genes (DEGs) and enrichment analysis

The sc.tl.rank_genes_groups function implemented in Scanpy was used to calculate DEGs among different clusters. The *t*‐test was performed on each gene, and the adjusted *p*‐value for statistical significance was computed. Adjusted *p* values <0.01 were considered as signature genes. We annotated cell clusters according to the expression of those signature genes reported in the literature. Gene ontology (GO) and Kyoto encyclopaedia of genes and genomes (KEGG) enrichment were performed on DEGs by clusterProfiler (v4.2.2) R package with a hyper‐geometrical statistical test with a threshold of 0.05.

### 2.7 Pseudotime trajectory construction

The trajectory analysis was performed using the Monocle2 R package (v2.22.0) to reveal cell differentiational fates in OAF cell clusters. We used dispersion table function to calculate the DEGs and sorted the cells into pseudo‐time order. Dimensional reduction and cell ordering were performed using the DDRTree method and the orderCells function.

### 2.8 Intercellular interaction analysis

To reveal intracellular communication networks, CellChat (v1.5.0) was applied for potential ligand–receptor analysis. Interaction pairs with *p*‐value <0.05 were considered significant and kept. The interactions between endothelial cells and chondrocytes were further analysed.

### 2.9 Immunohistochemical and immunofluorescence staining

Sampling fixations was performed using 4% paraformaldehyde for 7 days and processed with decalcification by EDTA for 30 days. Fixed samples were embedded with paraffin and sectioned into 6‐mm thick slices. Staining was performed using anti‐TGFBR3 antibody (1:200, 2000‐1‐AP, Proteintech), anti‐EGLN3 antibody (1:500, 55,398–1, Sabbiotech) and anti‐GPRC5A antibody (1:200, 10,309‐1‐AP, Proteintech). Images were scanned by a high‐resolution digital slide scanner (VS‐200, Olympus). For immunofluorescence staining, sections were incubated with proteinase K (1:200, Solarbio) for 30 min, subsequently with Triton X‐100 (0.1%, Beyotime) for 30 min, and with 3% calf serum for 30 min. The primary antibodies, including anti‐PDGFRA (1:200, PB0822, BOSTER) and anti‐PROCR (1:1000, ab56689, Abcam), were used to incubate sections at 4°C overnight, and then the secondary antibodies (1:500, A11008, A21244, A21422, Invitrogen) were applied for 1 h at room temperature. Nuclei were stained with DAPI solution (0.1%, Beyotime). The immunostaining image was captured by the fluorescence microscopy system (CX43, Olympus) and analysed by the matching cellSens Dimension software (FCSnap) and Zen 2.3 (ZEISS).

### 2.10 Statistics analysis

Statistical analysis was performed with R (v4.1.3) and Python (v3.8.10) softwares. Differences with *p* value <0.05 were considered to be statistically significant.

## 3 RESULTS

### 3.1 Integrated analysis revealed the cellular heterogeneity in goat healthy and degenerative IVDs

**FIGURE 1:**
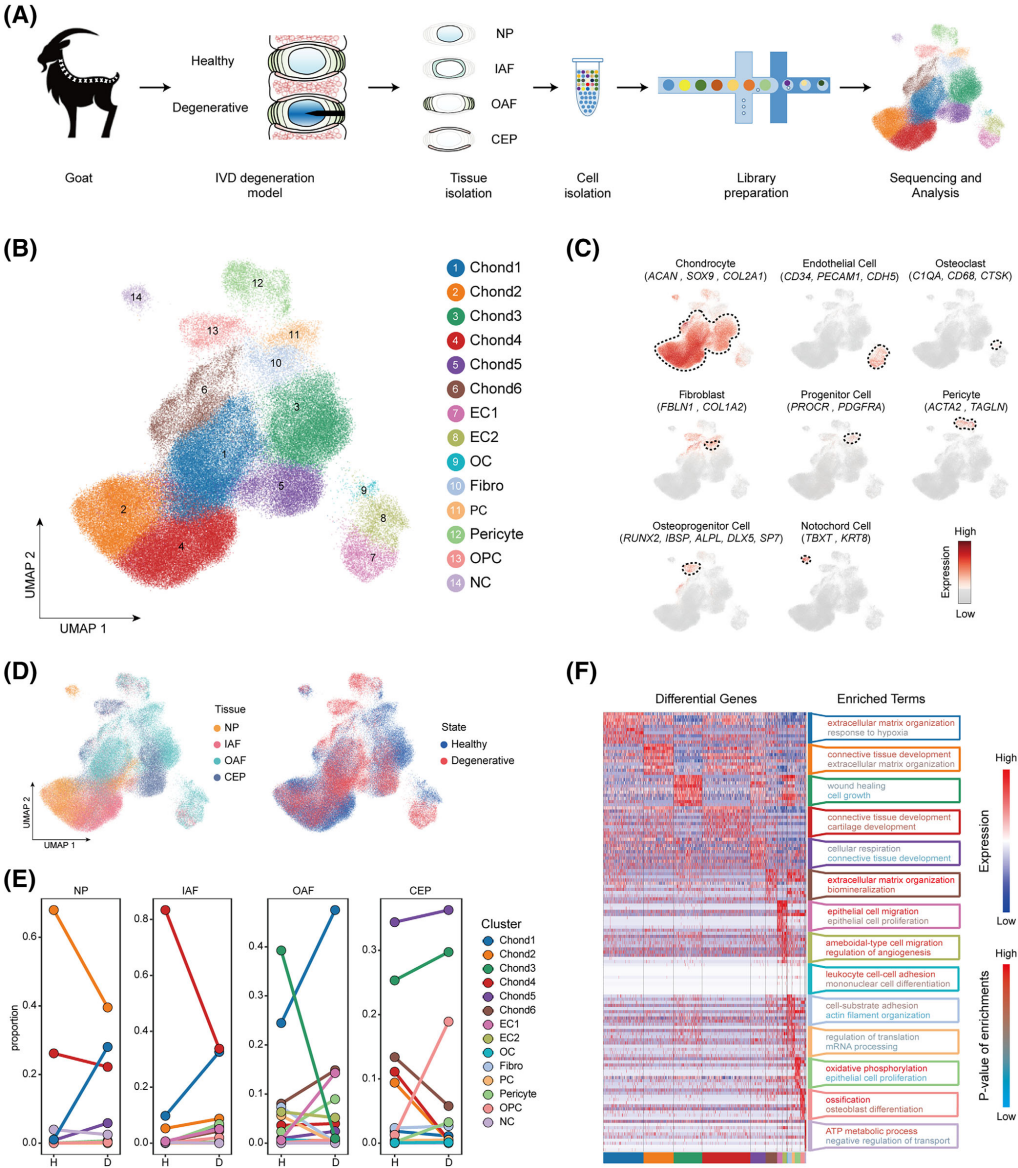
Single‐cell transcriptomic profiling of goat IVD cells. (A) Schematic workflow of the experimental strategy. Degenerative mode was established by acupuncturing IVD. Cells isolated from NP, IAF, OAF, and CEP of goat IVD were subjected to droplet‐based scRNA‐seq. (B) Distribution of 120,663 cells from goat IVDs. Fourteen clusters were visualised by UMAP plot. (C) The average expression of feature genes for clusters defined in (B) on the UMAP map. (D) UMAP plot of IVD cells, color‐labeled by anatomical regions and states. (E) Slope plots showing the spatiotemporal proportion of all clusters. (F) Heatmap and box showing the scaled expression of DEGs and enriched terms for each cell cluster. CEP, cartilage endplate; Chond, chondrocyte; D, degenerative; DEGs differentially expressed genes; EC, endothelial cell; Fibro, fibroblast; H, healthy; IAF, inner annulus fibrosus; IVD, intervertebral disc; NC, notochord cell; NP, nucleus pulposus; OC, osteoclast; OAF, outer annulus fibrosus; OPC, osteoprogenitor cells; PC, progenitor cell; scRNA‐seq, single‐cell RNA sequencing; UMAP, uniform manifold approximation and projection.

Goat IVDs were injured with a 4.5‐mm drill bit to build the model of IDD. MRI evaluated the degenerative degree of IVDs by Pfirrmann grading scales (Figure S1a).^21^ To decode the cellular heterogeneity of IDD, we performed droplet‐based single‐cell transcriptomic profiling (10× Genomics Chromium System) of cells from healthy (Pfirrmann I) and degenerative (Pfirrmann V) goat IVDs (Figure 1A). A total of 120,663 individual cells meeting the criterion of quality control were profiled (Figure S1b,c), including 69,683 cells from healthy samples (14,492 NP cells, 17,559 IAF cells, 29,101 OAF cells, and 8531 CEP cells) and 50,980 cells from degenerative samples (12,117 NP cells, 14,602 IAF cells, 13,659 OAF cells, and 10,602 CEP cells).

BBKNN integration was performed to remove the batch effects, and the UMAP illustrated 14 putative clusters in IVDs, including six clusters of chondrocytes (Chond1‐6), two clusters of endothelial cells (EC1 and 2), osteoclasts (OC), fibroblasts (Fibro), progenitor cells (PC), pericytes, osteoprogenitor cells (OPC), and notochord cells (NC) (Figure 1B). Chondrogenic transcription factor *SOX9* and cartilage matrix specific markers *ACAN* and *COL2A1* were expressed in chondrocytes (Figure 1C).^5^ We identified EC1/2 by expression of feature genes including *CDH5*, *CD34* and *PECAM1*, pericytes by *ACTA2*, *MYH11* and *TAGLN* (Figure 1C).^25^, ^26^, ^27^, ^28^, ^29^ *FAP*, *FBLN1* and *COL1A2* were specifically expressed in Fibro (Figure 1C).^30^, ^31^ OC and OPC were identified by specific feature genes (*C1QA*, *CD68* and *CTSK* for OC, *SP7*, *RUNX2*, *DLX5*, *ALPL* and *IBSP* for OPC) (Figure 1C).^32^, ^33^, ^34^ Notochord‐derived markers *TBXT* and notochord‐derived cytokeratin genes, such as *KRT8* were detected in NC (Figure 1C).^35^, ^36^ Specifically, a cluster of PC expressing *PROCR* and *PDGFRA* was identified, consistent with our previous findings in human IVDs (Figure 1C).^10^ We also compared the transcriptomic similarities between goat and human IVDs, and general conservation was observed among cell clusters (Figure S1e). The proportion of Chond1,5, EC1 and OPC increased in IDD while the proportion of Chond2,3,4, EC2 and OC decreased, implying the possible response of these clusters to IDD (Figure 1D,E).

We identified the DEGs among clusters and performed GO enrichment analysis (Figure 1F). Generally, all chondrocytes except Chond3 enriched terms related to extracellular matrix (ECM) organisation and connective tissue development. Specifically, Chond1 was sensitive to hypoxia, Chond3 was associated with wound healing and cell growth, Chond4 was active in cartilage development and Chond6 participated in biomineralisation‐related activities. EC1 was associated with EC migration and proliferation while EC2 was associated with angiogenesis. Leukocyte cell–cell adhesion and mononuclear cell differentiation were enriched in OC. Fibro showed features of cell–substrate adhesion and actin filament organisation. PC was active in the regulation of translation and mRNA processing. OPC was associated with ossification and osteoblast differentiation. Pericyte and NC both had high metabolic activities. Together with the analysis above, we illustrated the transcriptomic features of cell clusters in healthy and degenerative goat IVDs.

### 3.2 Intrinsic signatures in chondrocytes associated with IDD

**FIGURE 2:**
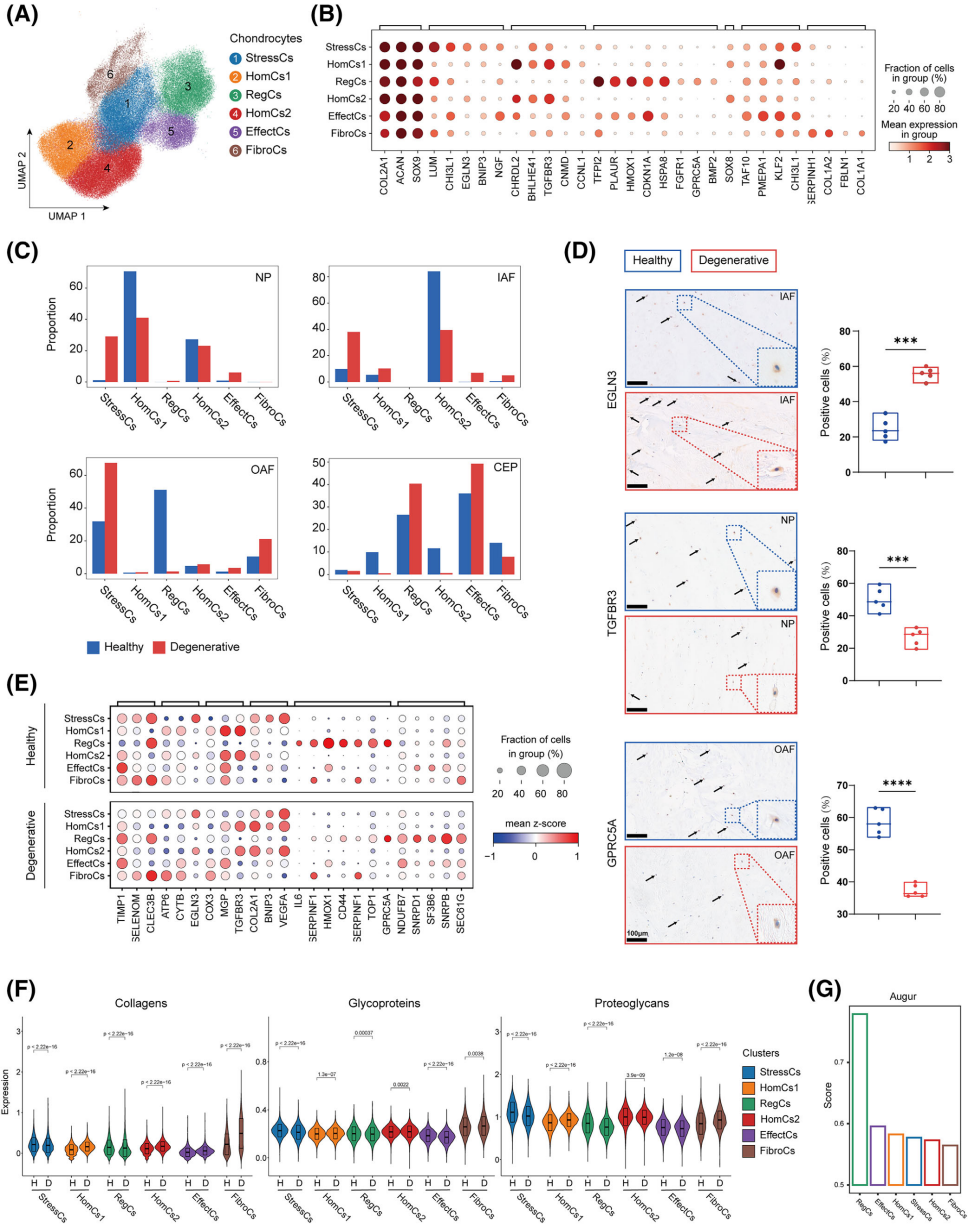
Identification of chondrocyte clusters and gene signatures in IDD. (A) 103,431 chondrocytes were embedded in UMAP plot with annotated names. (B) Dot plot displaying feature genes of each chondrocyte clusters. (C) Proportion of chondrocytes in NP, IAF, OAF, and CEP. (D) Immunohistochemistry staining of EGLN3, TGFBR3, and GPRC5A and the quantification of positive cells displayed in box plots (*n* = 5). Scale bar, 100 μm. (E) Dot plot showing DEGs between healthy and degenerative chondrocytes. (F) Violin plots showing the performance of gene set associated with core matrisome. (G) Augur score of each chondrocyte clusters. CEP, cartilage endplate; EffectCs, effect chondrocytes; FibroCs, fibrochondrocytes; HomCs, homeostatic chondrocytes; IAF, inner annulus fibrosus; IDD, intervertebral disc degeneration; NP, nucleus pulposus; OAF, outer annulus fibrosus; RegCs, regulatory chondrocytes; StressCs, stressed chondrocytes; UMAP, uniform manifold approximation and projection.

To decipher transcriptomic changes in chondrocytes of IDD, we identified Chond1‐6 as stressed chondrocytes (StressCs), homeostatic chondrocytes 1 (HomCs1), regulatory chondrocytes (RegCs), homeostatic chondrocytes 2 (HomCs2), effect chondrocytes (EffectCs) and fibrochondrocytes (FibroCs) (Figure 2A and Figure S2a). DEGs were found among the six chondrocyte subclusters (Figure 2B). StressCs specifically expressed Egl‐9 family hypoxia inducible factor 3 (*EGLN3*) related to cellular tolerance to hypoxia stress and ECM genes *LUM* and *CHI3L1*, implying this subcluster participated in the synthesis and regulation of ECM.^37^ The homeostatic chondrocytes have been reported with the expression of *CCNL1*.^10^ We found that both HomCs1 and HomCs2 expressed rhythmic genes, such as *BHLHE41*, showing their maintenance of homeostasis by controlling the cell rhythms.^38^ *TGFBR3*, the important receptor in the TGF‐β signal pathway that contributes to the growth and development of chondrocytes, is highly expressed in HomCs1 and 2.^39^ RegCs expressed *BMP2*, *HMOX1* and *GPRC5A*.^10^ *HMOX1* protects chondrocytes from ageing induced by hypoxia stress and *GPRC5A* promotes cell proliferation.^40^, ^41^ EffectCs expressed transcription initiation factor *TAF10*. FibroCs were marked by the expression of *COL1A1*, *COL1A2* and fibrosis‐related genes, such as *SERPINH1* and *FBLN1*.^15^

We found StressCs increased significantly in degenerative NP, IAF, and OAF, which was validated by immunohistochemical staining of StressCs marker EGLN3 (Figure 2C,D). DEGs and GO analysis showed that StressCs in healthy IVDs enriched RNA splicing, protein maturation, and highly expressed connective tissue development‐related genes *TIMP1* and *SELENOM* (Figure 2E and Figure S2b). However, in IDD, StressCs responded strongly to oxidative stress and external stimulus, proceed metabolism process and wound healing, and highly expressed ATP metabolic process related genes *ATP6*, *CYTB*. StressCs had a high score in collagens synthesis and low scores in glycoproteins and proteoglycans synthesis (Figure 2F). These results showed StressCs resisted the hypoxia environment in IVDs induced by degeneration. On the contrary, HomCs decreased in IDD, which was validated by immunohistochemical staining of TGFBR3 (Figure 2C,D). In particular, healthy HomCs showed high metabolic activity, cellular respiration, and expressed cartilage development‐related genes *MGP* and *EGR1*, while degenerative HomCs exhibited catabolic process, ossification, regulation of neurogenesis and epithelial cell proliferation, with higher expression of *VEGFA* (Figure 2E, Figure S2b). HomCs became more active in collagens and glycoprotein synthesis in IDD (Figure 2F). Similar to HomCs, RegCs decreased in degenerative OAF, which is validated by immunohistochemical staining of GPRC5A (Figure 2C,D). We performed Augur analysis, a method to prioritise the cell types most responsive to biological perturbations in single‐cell data, and identified RegCs as the most sensitive chondrocyte clusters in response to degeneration (Figure 2G).^42^ In particular, RegCs expressed highly collagens‐related genes in IDD (Figure 2F). Collectively, we found RegCs took collagens synthesis as the major function but lost repairing ability in IDD.

We also analysed the transcriptomic changes across anatomy sites (Figure S2c,d). Specifically, ATP metabolic process was enriched in healthy NP and IAF except for OAF. Degenerative NP, IAF and OAF enriched ECM organisation. Chondrocytes in degenerative NP and IAF maintained their homeostasis by regulation of apoptotic signalling pathway and were associated with epithelial cell migration‐related activities, suggesting that NP and IAF were the more severely damaged in IDD (Figure S2c,d).

In summary, we found that StressCs increased significantly in NP, IAF and OAF with higher expression of oxidation stress‐related genes while HomCs decreased in NP and IAF with impaired homeostasis maintaining ability, and RegCs decreased in OAF with the stronger function of collagens synthesis. These results revealed the transcriptomic spatiotemporal variability of chondrocytes in IDD, implying the phenotype of chondrocytes switched from maintaining homeostasis to resisting stress and exhaustion of repairing capacity.

### 3.3 Switched differentiation programs of PCs and stemness exhaustion in NCs in IDD

**FIGURE 3:**
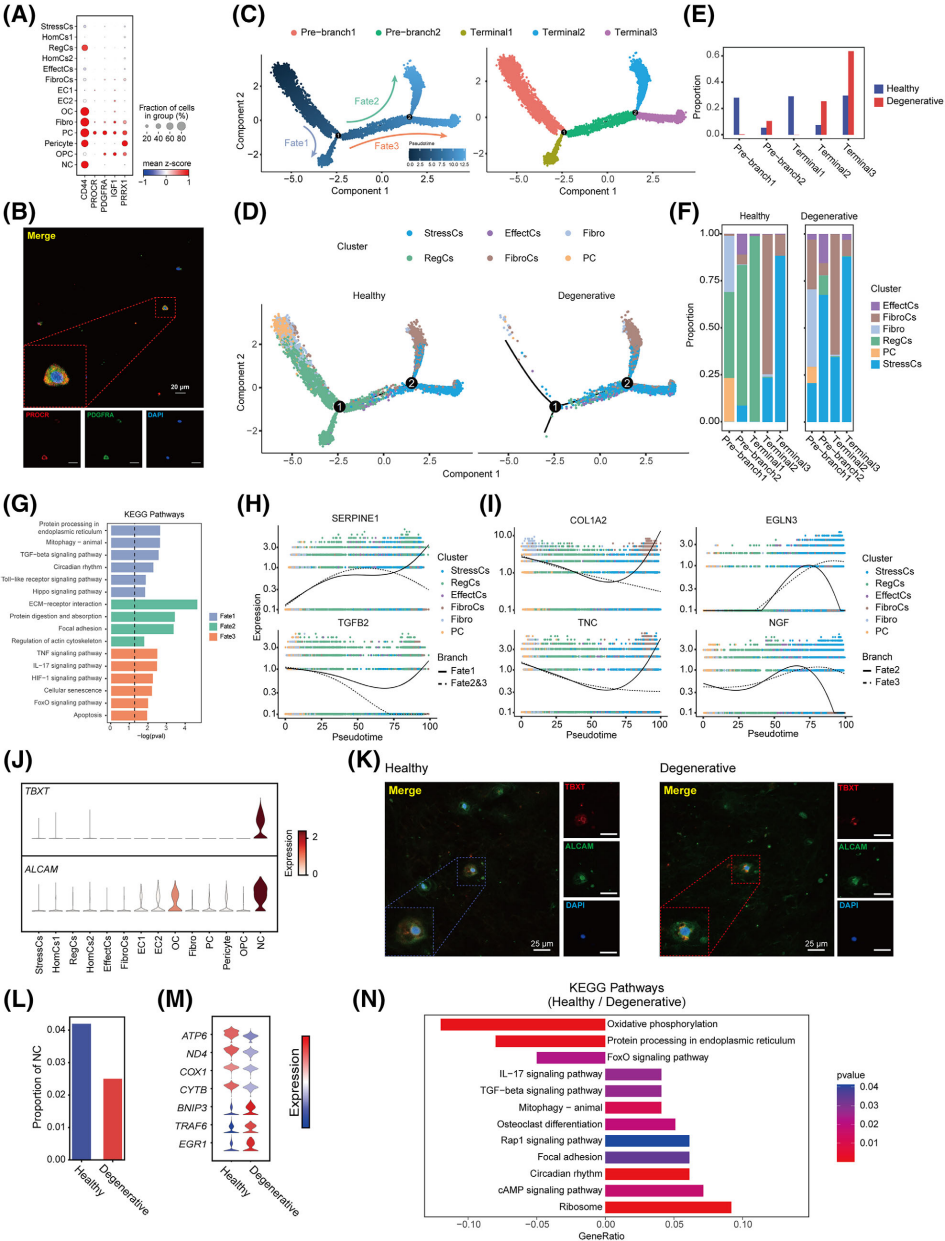
Differentiation trajectories of progenitor cells (PCs) and Notochord cells (NCs) in IDD. (A) Dot plot showing the feature genes of PC. (B) Immunofluorescence staining showing the co‐expression of PROCR and PDGFRA in goat IVDs (*n* = 3). Scale bar, 20 μm. (C,D) Reconstruction of bilineage trajectories in OAF cells by Monocle2. Three fates were established and PC lied in the root of the trajectory. (E) Proportion of total cells in different parts in (C). (F) Proportion of different cell clusters in different parts in (C). (G) Enriched KEGG pathways in different differential fates. (H,I) Fitting curves showing the trend of key molecules in different fates. (J) Violin plot showing the specific expression of *TBXT* and *ALCAM* (CD166) in NCs. (K) Immunofluorescence staining showing the co‐expression of TBXT and ALCAM in goat IVDs (*n* = 3). Scale bar, 25 μm. (L) Histograms showed the NCs numbers in healthy and degenerative IVDs. (M) Violin plot showing DEGs between healthy and degenerative NCs. (N) Histogram showing enriched KEGG pathways for healthy and degenerative NCs. IDD, intervertebral disc degeneration; IVDs, intervertebral discs; KEGG, Kyoto encyclopedia of genes and genomes; OAF, outer annulus fibrosus.

*PROCR* ^+^ PCs specifically expressed *PDGFRA*, *CD44*, *IGF1* and *PRRX1*, potential stemness‐related genes in IVDs (Figure 3A).^10^ GO analysis showed PCs enriched mesenchyme development, mesenchymal cell differentiation, and stem cell differentiation functions (Figure S3a). With the highest Augur score, PCs were suggested to be the most sensitive cluster to IDD (Figure S3b). Immunofluorescence staining of PROCR and PDGFRA proved their existence in goat OAF, different from their location in human IVDs (Figure 3B).^10^ To analyse the alteration in chondrogenic potential of *PROCR* ^+^ PCs of IDD, we simulated the chondrogenic trajectory by Monocle2 (Figure 3C,D). PCs lied in the root of trajectory and displayed three differentiation fates to RegCs, FibroCs and StressCs respectively (Figure 3C,D). We uncovered that the differentiation to RegCs (terminal1) almost disappeared, while differentiation to StressCs (terminal3) took the dominant position and StressCs became the major population in IDD (Figure 3E,F).

To analyse the signature alteration along the differentiation trajectory, DEGs of three fates were used for KEGG analysis (Figure 3G). The results showed that cells in fate1 had high activity in protein synthesis, mitophagy, and circadian rhythm. Interestingly, we found TGF‐β signal pathway and Hippo signal pathway were enriched in fate1 (Figure 3G,H). Both pathways were associated with the formation of cartilage, implying their potential of differentiation to RegCs.^39^, ^43^ In fate2, ECM‐receptor interaction and focal adhesion were enriched, with highly expression of *COL1A2* and *TNC* (Figure 3G,I). Notably, TNC was proven to induce adhesion and modulate the maintenance of PCs of IVDs.^11^ Thus, *COL1A2* and *TNC* could be the key factors to indicate the fate to FibroCs. Fate3 enriched TNF and IL17 signalling pathways (Figure 3G,I). TNF is an important proinflammatory factor, which leads to the damage of ECM in IVDs, cell ageing and autophagy, and the degeneration of IVDs.^44^, ^45^ Notably, HIF‐1 signalling pathway was also enriched in fate3 (Figure 3G). HIF‐1 pathway is induced in cell apoptosis and autophagy, to maintain cellular homeostasis in hypoxia stress.^46^, ^47^ By comparing the molecular features of PC differentiational trajectories in healthy and degenerative IVDs, we found hypoxia related genes such as *EGLN3*, nerve growth related genes *NGF* were upgraded in fate3 in IDD while the expression of *SERPINE1*, *TGFB2*, *COL1A2* and *TNC* showed less variation (Figure S3c).^37^, ^48^ Therefore, *EGLN3* and *NGF* might be the important factors leading to the shift in chondrogenic differentiation of *PROCR* ^+^ PCs in IDD.

Notochord is the important origin of NP cells during embryo and post‐natal development.^49^ Our study uncovered that NCs specifically expressed the known markers such as *TBXT*, *KRT8* and a novel marker *ALCAM* (Figure 3J). Immunofluorescence staining of TBXT and CD166 (encoded by *ALCAM*) showed two molecules were highly co‐expressed, suggesting the potential roles of ALCAM as the novel marker of notochord cells (Figure 3K). Importantly, the number of NCs decreased in IDD (Figure 3L). We compared the DEGs between healthy and degenerative NCs. The results showed that oxidative phosphorylation‐related genes such as *ATP6*, *ND4* and *COX1* and regeneration‐related genes such as *CYTB* decreased while mitophagy‐related genes *BNIP3*, *IL17* signal pathway‐related genes *TRAF6* were increasingly expressed in IDD (Figure 3M). KEGG and GO analysis showed oxidative phosphorylation, protein processing in the endoplasmic reticulum and ATP metabolism were enriched in healthy NCs, while IL17 signal pathway, mitophagy and response to decreased oxygen levels were enriched in degenerative NCs (Figure 3N, Figure S3d). Collectively, the response to inflammation through mitophagy possibly resulted in NCs decreasing and exhaustion in differentiation function.

In summary, we found that the differentiation fate of PCs was switched and NCs showed stemness exhaustion, which should be the cause for their loss of self‐healing ability in IDD.

### 3.4 Identification of *CAV1* ^+^ endothelial cells penetrated in degenerative IVDs

**FIGURE 4:**
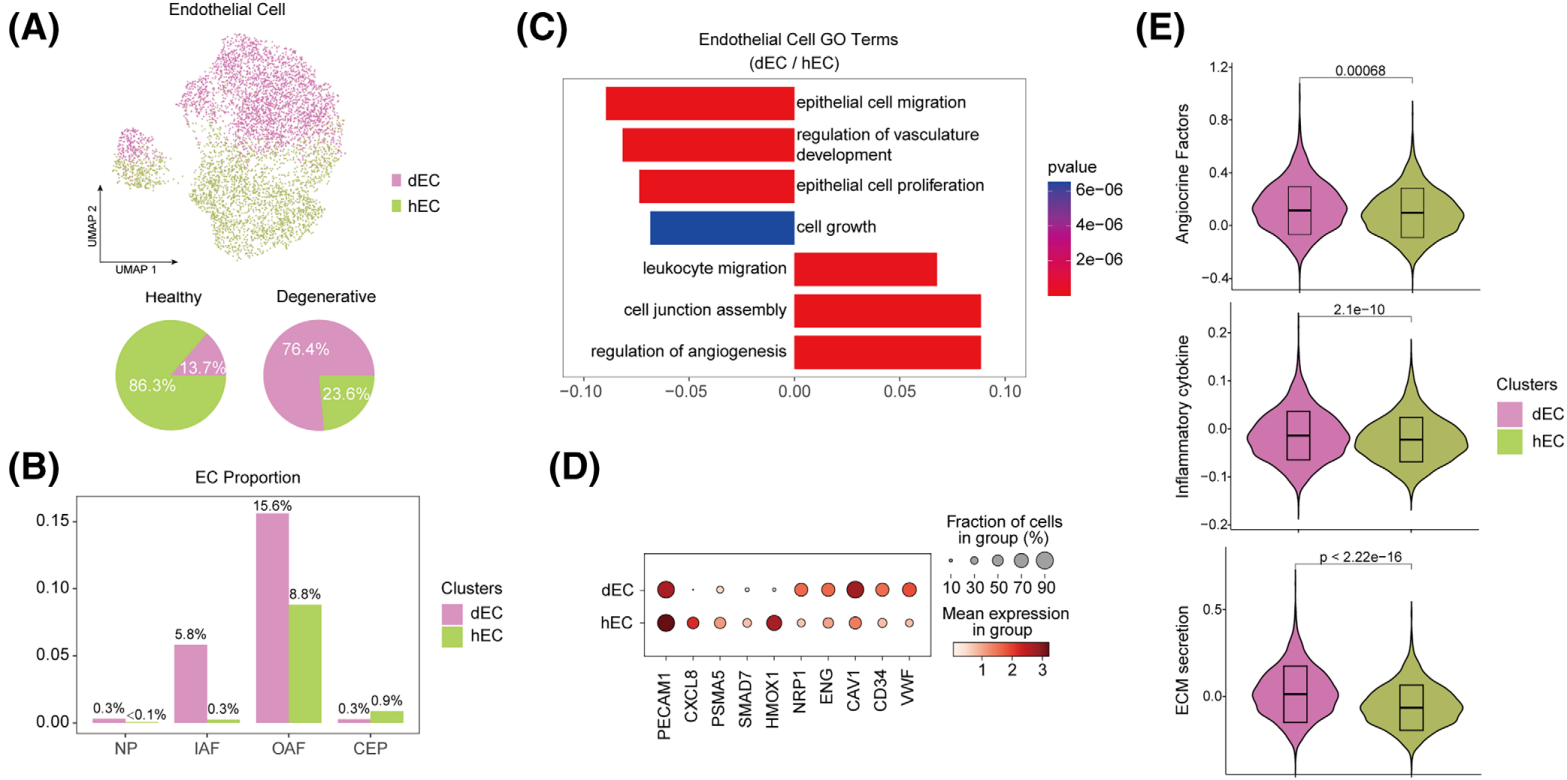
Alteration of endothelial cell phenotype in IDD. (A) UMAP plot of two clusters of 5738 endothelial cells identified in IVD. The pie charts showed proportion of hEC or dEC in all endothelial cells. (B) Histograms showing the proportion of hEC or dEC in different tissue of IVD. (C) Histograms showing the enriched GO terms in dEC and hEC. (D) Dot plot showing the DEGs between dEC and hEC. (E) Violin plots showing the scores of gene set related to endothelial functions. dEC endothelial cells in degenerative IVD; GO, gene ontology; hEC, endothelial cells in healthy IVD; IDD, intervertebral disc degeneration; IVDs, intervertebral discs; UMAP, uniform manifold approximation and projection.

Previous studies have shown that vessels invade IVDs during DDD progression, leading to pain and dysfunction in the disc.^50^, ^51^ We found EC1 was the dominant ECs in degenerative IVDs thus named as degenerative EC (dEC), while EC2 was the major ECs in healthy IVD, thus named as healthy EC (hEC) (Figure 4A). Interestingly, hECs were located in OAF, while dECs were located both in IAF and OAF (Figure 4B). DEGs and GO analysis were performed between dECs and hECs (Figure 4C,D). dECs specifically expressed *CAV1*, *VWF* and *ENG*, among which *CAV1* is associated with endothelial cell migration, and modulates cell responses through the force‐dependent structure of the microenvironment.^52^, ^53^ In addition, CAV1 is the marker of type E endothelial cells which strongly supports osteoblast lineage cells and later gives rise to endothelial cell subpopulations.^54^ These data suggested that ECs penetrated the degenerated IVDs under long‐term high loading stress. GO analysis showed dECs were associated with endothelial cell migration and proliferation, and regulation of vasculature development (Figure 4C). Furthermore, we found that dECs displayed higher score in angiocrine factors, inflammatory cytokine and ECM secretion (Figure 4E).

**FIGURE 5:**
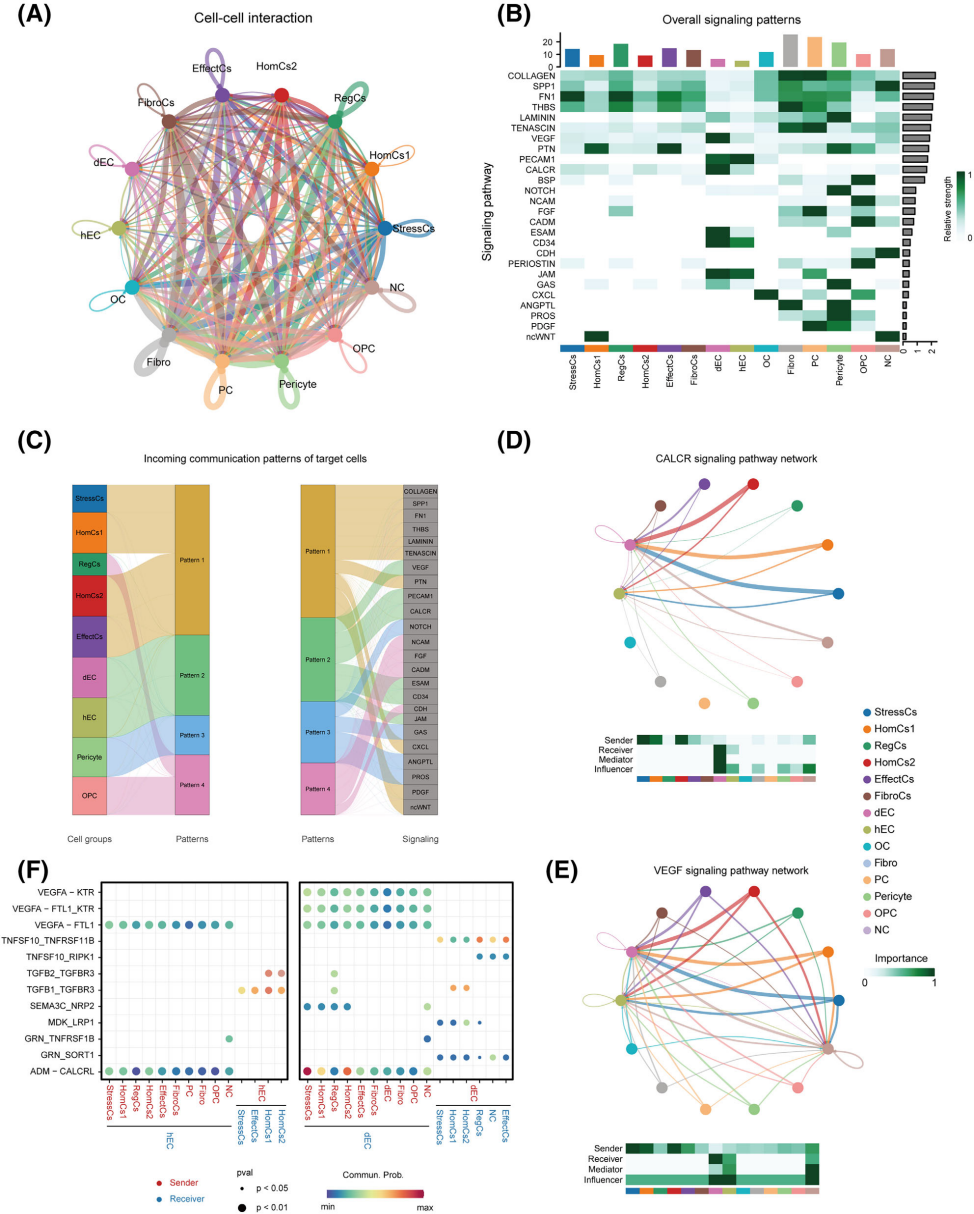
Crosstalk networks of cell clusters in IVD. (A) Overview of the cellular network regulating the homeostasis of IVD. Width of curves indicate the crosstalk strength. (B) Heatmap showing the signalling pathways that each cluster participated in. Histogram calculated the number of the pathways that clusters participated in (upper) and clusters that the pathways enriched in (right). (C) River plot showing the incoming pattern of each cluster and the pathways in each pattern. (D,E) Circle plot showing the inferred CALCR (D) and VEGF (E) signalling networks. (F) Dot plot showing the communication probability of the indicated ligand‐receptor pairs between ECs and other cell clusters. IVDs, intervertebral discs.

In order to analyse the functions of EC penetration, we established cellular communication networks in cell clusters. CellChat analysis revealed the complex cell–cell interaction among IVD cell clusters, and ECs enriched lots of signalling pathways such as VEGF, PECAM1 and CALCR (Figure 5A,B, Figure S4a). We further analysed the communication patterns of ECs (Figure 5C, Figure S4b). The incoming pattern in ECs were classified into pattern2 including VEGF and CALCR signalling pathways (Figure 5C, Figure S4b). CALCR signalling pathway is proven to promote vessel formation and protect vessel function.^55^ VEGF signalling pathway can induce endothelial infiltration and vasculogenesis.^56^ Intriguingly, by analysing the roles of all clusters in CALCR and VEGF pathways, we found significantly different patterns between dECs and hECs, that the major signal receiver was dECs but not hECs (Figure 5D,E). Meanwhile, StressCs and HomCs were the major signal senders of CALCR and VEGF pathways, which implied these clusters might induce EC penetration in IDD (Figure 5D,E). CALCR pathway includes ADM‐CALCRL pair and VEGF pathway includes VEGF‐KDR, VEGF‐FTL1 and VEGF‐FTL1_KDR pairs (Figure S4c,d). In addition, we uncovered that chondrocytes secret *SEMA3C* as a potential factor to inactivate dEC (Figure 5F).^57^ dECs secreted *TNFSF10* to chondrocytes, leading to the impairment of chondrocyte function and inducing the degeneration of IVDs (Figure 5F).^58^ These results revealed the regulatory networks between dECs and chondrocyte clusters.

In brief, we speculated that cellular crosstalk among these clusters could result in EC penetration, while ECs secreted cytokines to negatively regulate chondrocyte functions in IDD. Importantly, our study identified a *CAV1* ^+^ endothelial population, which will expand the understanding of vessel invasion and its role in the pathological process of IDD.

## 4 DISCUSSION

Previous studies have shown spheroidal and chondrocyte‐like cells lie in the inner zone of IVDs.^59^ And due to stress overload and lack of nutrient supply, these cells undergo substantial biological changes, including the alteration of cell composition and molecular phenotype during disc degeneration.^60^ Consistently, we found the proportions of various types of chondrocytes fluctuated in IDD (Figure 1). Briefly, StressCs increased tremendously while HomCs and RegCs decreased, indicating the chondrocyte phenotype switched into stress resistance and led to the failure of homeostasis in the degenerative disc.

Previously, we found *PECAM1* ^+^ ECs resident in the healthy human IVD.^10^ In goat, *PECAM1* ^+^ ECs located in the OAF of healthy IVD, while ECs increased and located in the IAF and OAF of degenerative IVD (Figure 1B–E). This finding was consistent with the previous studies that ectopic ingrowth of vessels into IVD secreted inflammation cytokines, promoted ECM degradation, and finally led to IDD.^50^, ^51^, ^61^ As capillary mural cells, pericytes played a role in stabilising newly formed blood vessels and expressed Tie2 controlling angiogenesis and vessel maturation.^62^, ^63^ *ACTA2* ^+^pericytes were firstly identified in healthy human IVD.^10^ In line with that, *ACTA2* ^+^ *TAGLN* ^+^ pericytes could also be detected in AF and CEP of healthy goat IVD, which increased in IDD (Figure 1B–E). We revealed that the pericytes was mainly increased in OAF and secondarily in CEP in degenerative IVDs, indicating the vascularization in IDD. In degenerative IVDs, OCs secrete inflammatory cytokines to promote the recruitment of immune cells, leading to inflammatory responses and neurotrophin release, which aggregate disc degeneration.^50^, ^64^ In our study, a cluster of *CTSK* ^+^ *CD68* ^+^ OCs was discovered in OAF, possibly migrated along the penetrated vessels, and involved in maintaining the homeostasis in IDD (Figure 1B–E).^65^ We found that *RUNX2* ^+^ *SP7* ^+^ OPCs distributed in CEP and increased in IDD, contributing to CEP osteogenesis and disruption of the nutrition supply for the inner IVDs (Figure 1B–E).^8^

In IDD, NP cells experience the destruction of mitochondrial structure and function,^66^, ^67^ while CEP cells turn to calcification.^68^ In this study, we revealed RegCs mainly located in OAF and CEP highly expressed growth factor *HMOX1* (encoding HO‐1), which induced autophagy protects against IL‐1β‐mediated apoptosis in human NP cells reflecting the repairing capacity to maintain the IVD homeostasis IVD (Figure 2B).^69^ Previous studies indicated the fissuring and neovascularization occurred in OAF during disc degeneration^7^, ^61^ Thus, the exhaustion of RegCs in OAF could facilitate the dysfunction in IDD (Figure 2C). We also found that HomCs decreased in the degenerated disc, reflecting the imbalance of chondrocytes homeostasis in disc degeneration (Figure 2C). Interestingly, we identified a population of *EGLN3* ^+^ StressCs with the potential function to resist hypoxia stress increased in NP, IAF and OAF in IDD (Figure 2B,C). PHD3 (encoded by *EGLN3*) is a transcriptional coactivator of HIF‐1α in NP cells,^70^ which suggested that StressCs may proliferate and resist the stress in degenerated disc.

Our previous study identified *PROCR* ^+^ PCs with potential stemness in human NP.^10^ Consistently, our scRNA‐seq analysis found *PROCR* ^+^ PCs were resident in goat IVDs with highly conservative transcriptomic characteristics (Figure 3A). Instead, goat IVD PCs locate in OAF, which is nearby the stem cell niches responsible for repairing activities,^71^, ^72^ suggesting the possible migration of these PCs from non‐NP regions induced by degeneration. However, PCs differentiate into StressCs rather than RegCs, indicating that chondrocyte functions switched from homeostasis maintenance towards stress resistance in degenerative IVDs (Figure 3C–F). Moreover, TNF and IL17 enriched in fate3 can promote chondrocytes to produce VEGF, and may induce endothelial cell invasion, exacerbating disc degeneration (Figure 3G).^73^

NCs can maintain the proteoglycan in IVD and protect NP cells from degradation and apoptosis.^74^ In this study, NCs were distinguished by previously identified biomarkers of *TBXT* and *KRT8*.^35^, ^36^ Interestingly, *ALCAM*, also known as *CD166*, was uncovered to be specifically expressed in NCs, which could be a potential novel biomarker of NCs (Figure 3J,K). ALCAM was one of the cell surface antigens associated with mesenchymal stem cells, which also implied the stemness of NCs.^75^ Previous study showed the loss of NCs in NP as the onset of the degenerative process.^76^ In this study, NCs in degenerated IVD showed high expression of mitophagy and inflammation‐related genes (Figure 3N). In brief, our findings suggested the co‐existence and divergent responses to the degeneration of PCs and NCs in IDD.

In this study, two clusters of ECs were discovered (Figure 4A). It is worth noting that the ECs showed an interesting spatiotemporal alteration in IDD, that was their spatial positioning, cell abundance and molecular characteristics turned to infiltration preference. Notably, *CAV1* ^+^ dECs were detected more in IAF, consistent with reported vascular penetration in IDD (Figure 4B). CAV1 can promote epithelial cell migration via FMNL2 formin and co‐ordinate with the microenvironment to enhance cell behaviours through the force‐dependent organization of the surrounding 3D environment.^52^, ^53^ Thus, CAV1 was possibly related to the migration of dEC and played an important role in cellular crosstalk under mechanical stress. VEGF is a classical inducer of angiogenesis and regulated vascularization in multiple tissues including degenerative disc.^56^, ^77^ Cell–cell interaction analysis showed VEGF played an important part in intercellular communication between ECs and other clusters (Figure 5E). In addition, CALCR promoted vascular formation and protected vessels,^55^ which was also enriched in the degenerated disc (Figure 5D). These results implied the potential roles of these pathways in vascularization in IDD. Previous studies showed that chondrocytes generated proinflammatory molecules leading to ectopic ingrowth of vessels in degenerated IVDs.^50^, ^78^ Consistently, we found StressCs and HomCs were the major signal senders of VEGF and CALCR pathways, suggesting their potential roles to induce vascularization in IDD (Figure 5D,E).

However, we did not detect nerve cells in our goat model of puncture‐induced degeneration. This could result from insufficient degeneration progression, although we observed a subset of vascular endothelial cells. Another possible explanation is the limitation of scRNA‐seq in capturing rare cell populations, as nerve cells may be even less frequent than endothelial cells in IVD.^79^ The goat model of puncture‐induced degenerative IVD mimics human disc degeneration more closely, but it requires further improvement for studying intrinsic disc degeneration.

**FIGURE 6:**
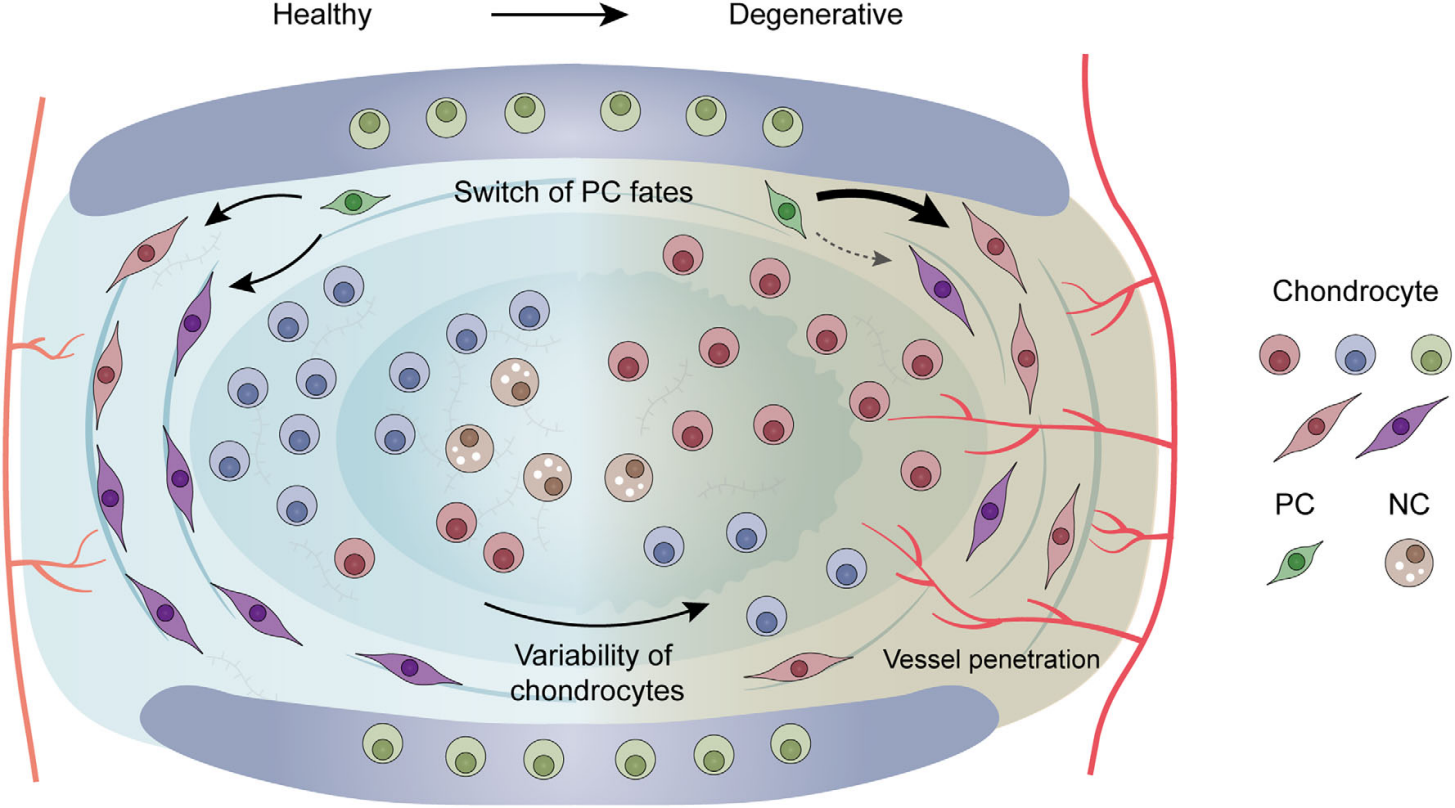
Graphical abstract of cell alteration in IDD. The key biological events of IDD were revealed by scRNA‐seq analysis, including variability of chondrocytes, switch of PC fates and vessel penetration. IDD, intervertebral disc degeneration; scRNA‐seq, single‐cell RNA sequencing.

In summary, the spatiotemporal single‐cell transcriptomic atlas of goat IVDs resolved cellular heterogeneity and variability induced by degeneration, providing insights into the molecular basis of transcriptomic responses to IDD and facilitating the in‐depth investigation of treatments of IDD (Figure 6).

## AUTHOR CONTRIBUTIONS

*Designed the study*: Peng Liu, Yibo Gan, and Jian He. *Research consultants*: Bing Liu, Lin Chen, and Jianhua Zhao. *Established the animal model and collected samples*: Yibo Gan, Jun Zhu, and Zhong Wang. *Prepared the cell isolation and scRNA‐seq*: Yibo Gan. *Analysed and interpreted the sequencing data*: Peng Lin and Jian He. *Performed the immunohistochemistry and immunofluorescence staining*: Pulin Yan, Sha Huang, Ou Hu, Huaijian Jin, Yangyang Li, and Liang Zhang. *Designed the figures*: Peng Lin, Jian He, and Yibo Gan. *Wrote the article*: Peng Lin, Yibo Gan, Jian He, and Peng Liu. All authors read and approved the final manuscript.

## FUNDING INFORMATION

This study was supported by grants from National Natural Science Foundation of China (82272507, 32270887 and 32200654), National Key Research and Development Program of China (2022YFA1103202), Postdoctoral Innovative Talent Support Program (BX20220397 and 2019–298), the Open Project of State Key Laboratory of Trauma, Burns and Combined Injury (SFLKF202201 and SKLYQ201902), and Training Plan of Talents' Innovation of Army Medical Center of PLA (2019CXJSB013).

## CONFLICT OF INTEREST STATEMENT

The authors declare that they have no competing interests.

## Supporting information


**Figure S1.** Sample information and data quality control, Related to Figure 1. (a) Representative magnetic resonance T2 imaging of healthy and degenerative IVD samples. (b) Violin plots showing the UMI and genes number in sequenced cells. (c) Violin plots showing gene number of each sample. (d) Heatmap showing the Pearson correlations among samples in healthy or degenerative IVDs. (e) Heatmap revealing the Pearson correlations between cell clusters in goat and human IVDs.

**Figure S2.** Transcriptomic characteristics of chondrocytes, Related to Figure 2. (a) Violin plot showing the expression of chondrocyte specific markers. (b) Enriched Gene Ontology (GO) biological processes terms in healthy and degenerative chondrocytes. (c) Dot plot showing the different enriched GO terms of chondrocytes at anatomic level. (d) Dot plot showing the DEGs of chondrocytes at anatomic level.

**Figure S3.** Characteristic features of progenitor cells in IVD, Related to Figure 3. (a) Dot plot showing the enriched GO terms in PCs. (b) Augur score of all clusters in IVD. PC showed the highest score. (c) Violin plots showing the key molecular changes in different parts of trajectories in IDD. (d) Histogram showing the enriched GO terms for healthy and degenerative notochord cells.

**Figure S4.** Crosstalk networks among the cell clusters in IVD, Related to Figure 5. (a) Dot plot showing the number of incoming or outgoing interaction strength of each cluster. (b) River plot showing the outgoing pattern of each cluster and the pathways each pattern containing. (c, d) Chord plot showing the major ligand and receiver pairs of CALCR (c) and VEGF (d) signal pathway. IVD intervertebral disc.

## Data Availability

The scRNA‐seq data and expression matrics generated in this study were deposited in the National Omics Data Encyclopedia (https://www.biosino.org/node/index) under the accession accession number OEP003834. All other data are available from the corresponding authors upon request.
