# Supplementary figures and images for: Spatially multicellular variability of intervertebral disc degeneration by comparative single‐cell analysis
Source: Cell Prolif. 2023 Apr 6;56(10):e13464. doi: 10.1111/cpr.13464 (PMC10542621; doi:10.1111/cpr.13464)

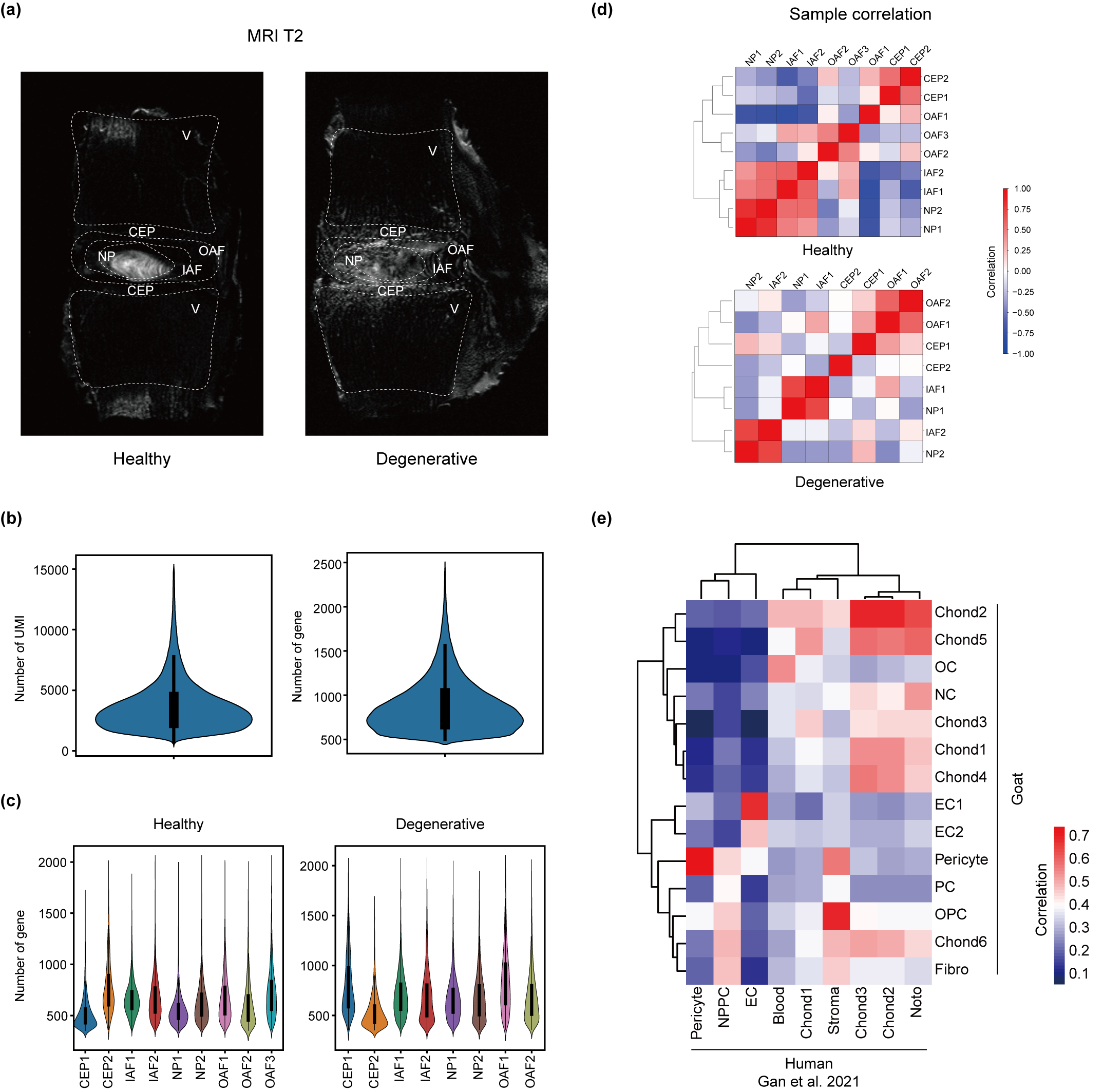

Supplement: Supplementary file 1 — Figure S1. Sample information and data quality control, Related to Figure 1. (a) Representative magnetic resonance T2 imaging of healthy and degenerative IVD samples. (b) Violin plots showing the UMI and genes number in sequenced cells. (c) Violin plots showing gene number of each sample. (d) Heatmap showing the Pearson correlations among samples in healthy or degenerative IVDs. (e) Heatmap revealing the Pearson correlations between cell clusters in goat and human IVDs. [file CPR-56-e13464-s002.tif]

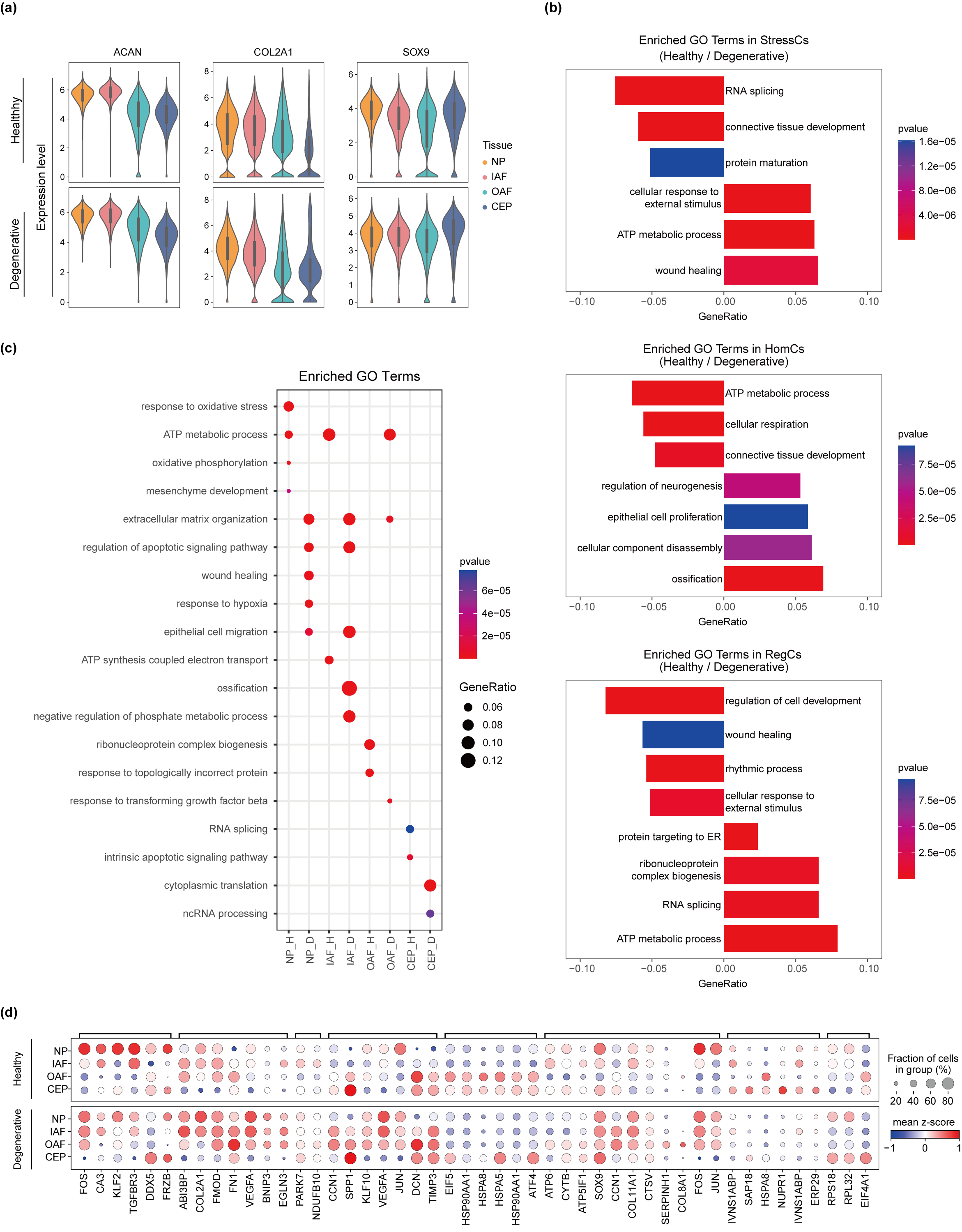

Supplement: Supplementary file 2 — Figure S2. Transcriptomic characteristics of chondrocytes, Related to Figure 2. (a) Violin plot showing the expression of chondrocyte specific markers. (b) Enriched Gene Ontology (GO) biological processes terms in healthy and degenerative chondrocytes. (c) Dot plot showing the different enriched GO terms of chondrocytes at anatomic level. (d) Dot plot showing the DEGs of chondrocytes at anatomic level. [file CPR-56-e13464-s001.tif]

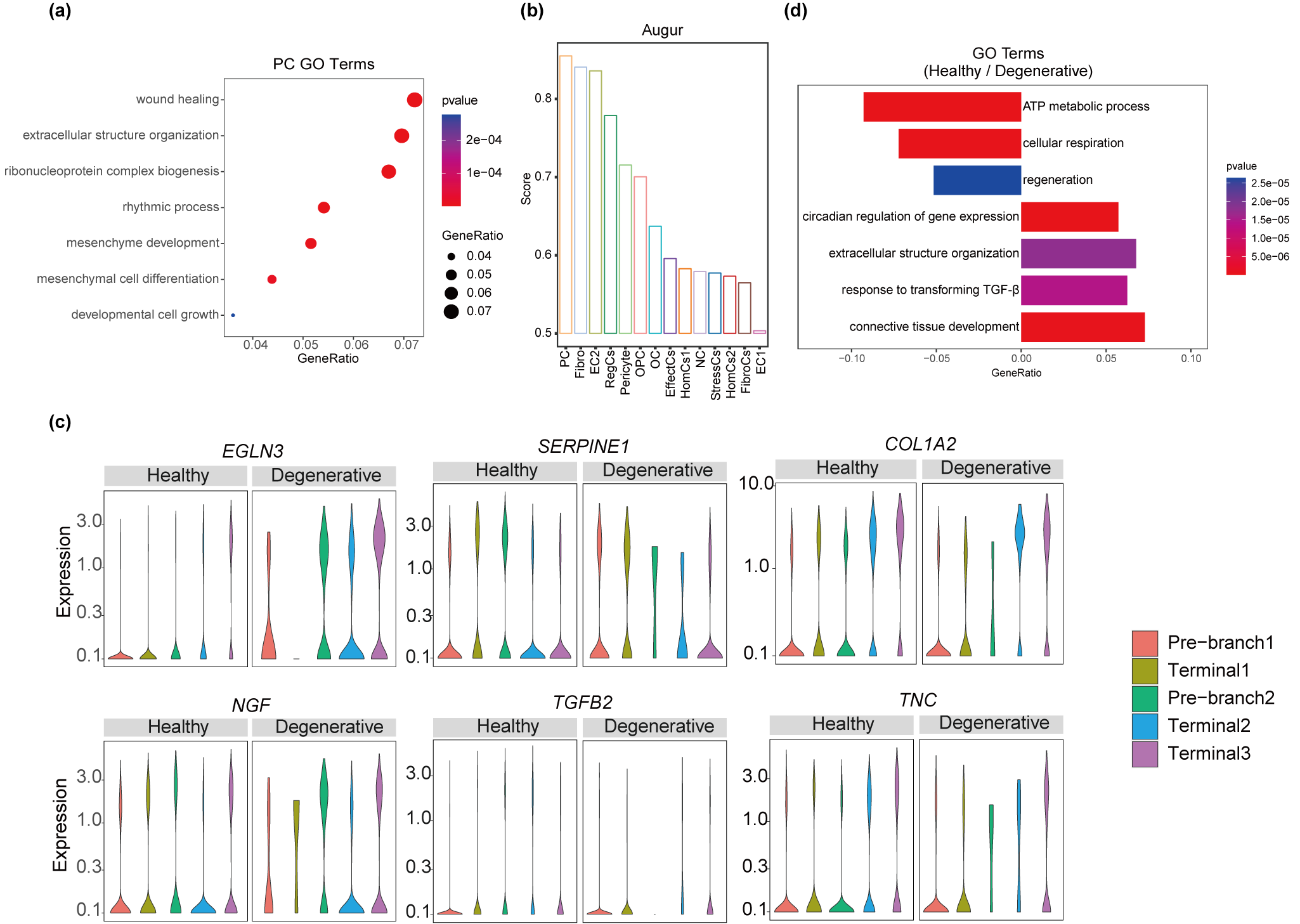

Supplement: Supplementary file 3 — Figure S3. Characteristic features of progenitor cells in IVD, Related to Figure 3. (a) Dot plot showing the enriched GO terms in PCs. (b) Augur score of all clusters in IVD. PC showed the highest score. (c) Violin plots showing the key molecular changes in different parts of trajectories in IDD. (d) Histogram showing the enriched GO terms for healthy and degenerative notochord cells. [file CPR-56-e13464-s003.tif]

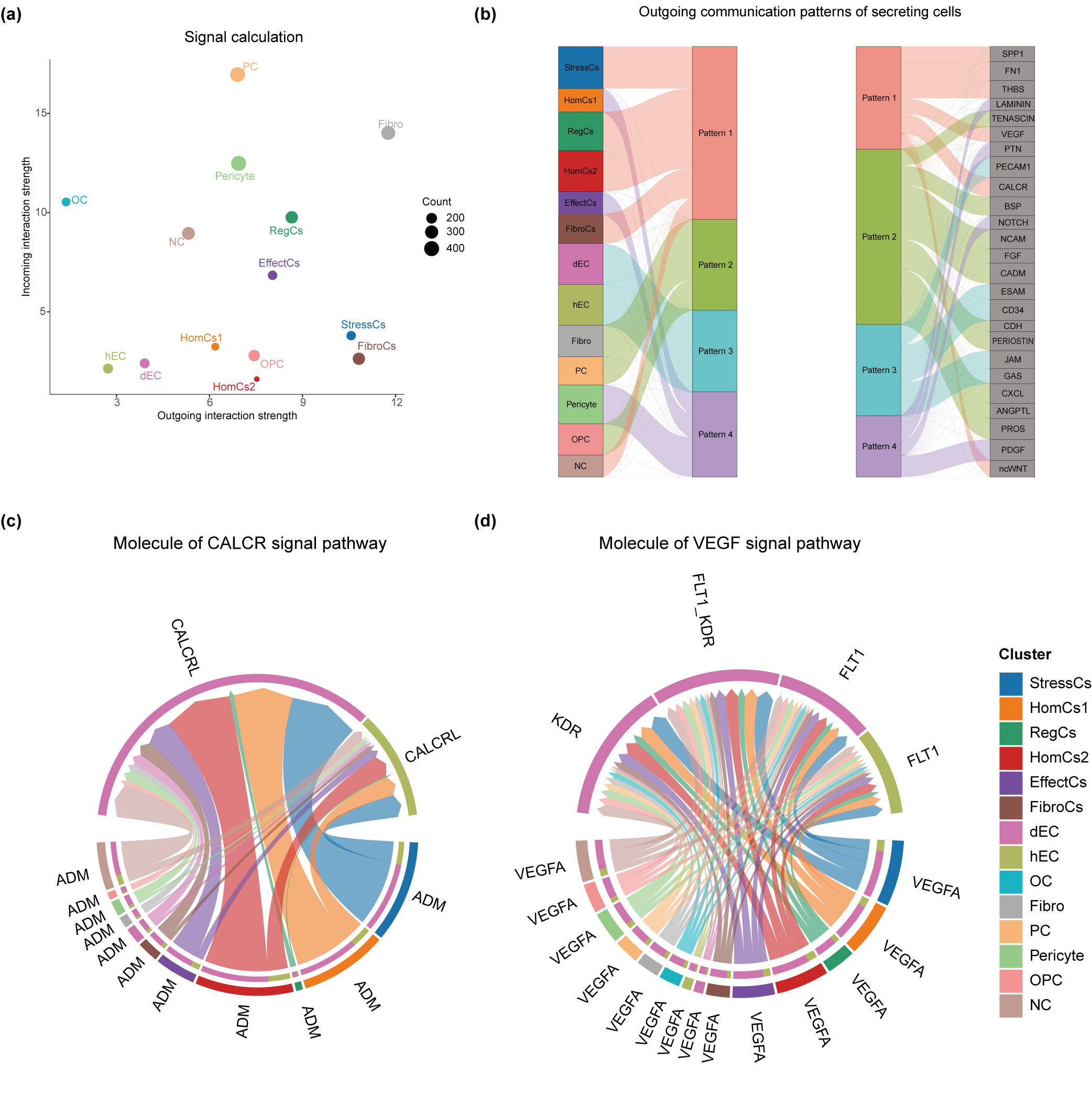

Supplement: Supplementary file 4 — Figure S4. Crosstalk networks among the cell clusters in IVD, Related to Figure 5. (a) Dot plot showing the number of incoming or outgoing interaction strength of each cluster. (b) River plot showing the outgoing pattern of each cluster and the pathways each pattern containing. (c, d) Chord plot showing the major ligand and receiver pairs of CALCR (c) and VEGF (d) signal pathway. IVD intervertebral disc. [file CPR-56-e13464-s004.tif]
